# The Role of Smoothened in Cancer

**DOI:** 10.3390/ijms21186863

**Published:** 2020-09-18

**Authors:** Kuo-Shyang Jeng, I-Shyan Sheen, Chuen-Miin Leu, Ping-Hui Tseng, Chiung-Fang Chang

**Affiliations:** 1 Division of General Surgery, Far Eastern Memorial Hospital, New Taipei City 22060, Taiwan; kevin.ksjeng@gmail.com; 2 Department of Hepato-Gastroenterology, Chang-Gung Memorial Hospital, Linkou Medical Center, Chang-Gung University, Taoyuan City 33305, Taiwan; happy95kevin@gmail.com; 3 Institute of Microbiology and Immunology, National Yang-Ming University, Taipei City 11221, Taiwan; cmleu@ym.edu.tw; 4 Institute of Biochemistry and Molecular Biology, National Yang-Ming University, Taipei City 11221, Taiwan

**Keywords:** Smoothened, Hedgehog signaling pathway, cancer stem cells

## Abstract

Smoothened (SMO) belongs to the Hedgehog (HH) signaling pathway, which regulates cell growth, migration, invasion and stem cells in cancer. The HH signaling pathway includes both canonical and noncanonical pathways. The canonical HH pathway functions through major HH molecules such as HH ligands, PTCH, SMO and GLI, whereas the noncanonical HH pathway involves the activation of SMO or GLI through other pathways. The role of SMO has been discussed in different types of cancer, including breast, liver, pancreatic and colon cancers. SMO expression correlates with tumor size, invasiveness, metastasis and recurrence. In addition, SMO inhibitors can suppress cancer formation, reduce the proliferation of cancer cells, trigger apoptosis and suppress cancer stem cell activity. A better understanding of the role of SMO in cancer could contribute to the development of novel therapeutic approaches.

## 1. Introduction

The Hedgehog (HH) signaling pathway is a conserved pathway involved in cell growth and tissue patterning [1,2,3,4,5]. It regulates tissue homeostasis and stem cell behaviors, but the pathway becomes quiescent in adult tissues. Abnormal HH signaling can be found in cancer of the skin, brain, liver, prostate and breast; in malignant blood disease; etc. [6].The canonical HH signaling pathway molecules includes Hedgehog ligands (Sonic hedgehog, Indian hedgehog and Desert hedgehog), PTCH (PTCH-1 and PTCH-2), Smoothened and GLIs (GLI-1, GLI-2 and GLI-3). In the non-canonical SHH signaling pathway, SMO or GLIs are activated by other pathways such as the mammalian target of rapamycin-protein kinase B (mTOR-Akt), protein kinase A (PKA) or Rho [1,2,3,4,5]. SMO is an important target in cancer treatment. The efficacy of SMO inhibitors for treatment of malignancies of the breast, liver, pancreas and colon cancer has been demonstrated or is under clinical trials [7,8,9]. However, SMO mutations may lead to resistance against SMO antagonists. Here, current studies pertaining to the oncopathogenic roles of SMO and its inhibitors in cancer therapy are reviewed.

## 2. Canonical Hedgehog Signaling Pathway

The canonical HH signal pathway molecules include HH ligands, Patched receptors, the Smoothened receptor (SMO) and GLI transcription factors [7]. In addition, suppressor of fused protein (SUFU) is a negative regulator of HH signaling. SUFU mutations have been identified to activate aberrant HH pathway in cancer HH ligands to bind to PTCH, and PTCH can thereby release the inhibition of SMO [10]. Three HH ligands (SHH, IHH and DHH) are involved in organ homeostasis and cell fate differentiation, and their expression are associated with cancer progression [11,12]. The activated SMO migrates to the cell plasma membrane and transduces signals to the nucleus via GLI proteins to turn on the expressions of target genes [1,2]. Its target genes are involved in cancer cell invasion, cell cycle, cell growth and stem cell activity. The aberrant activation of HH signaling pathway is associated with cancer development [13].

**Figure 1:**
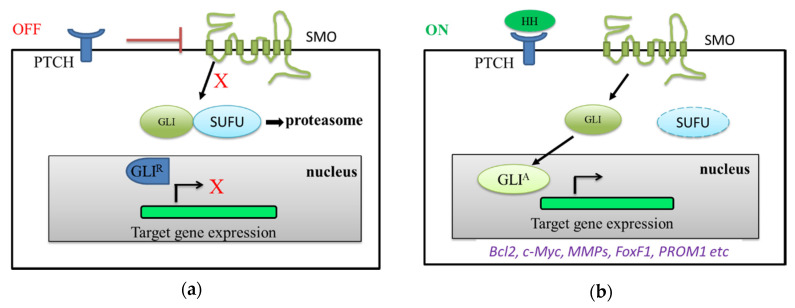
Canonical Hedgehog (HH) signaling pathway. (**a**) OFF state: PTCH inhibits Smoothened (SMO) activity, and transcription factor GLI and SUFU are proteolytic by proteasome. The GLI repressor form (GLI^R^) binds to target genes and there is no target gene expression. (**b**) ON state: HH ligands binds to PTCH to weaken the inhibition of SMO. SMO can then activate transcription factor GLI. The SUFU is removed from the binding to GLI. Therefore, GLI activator form (GLI^A^) to regulate target gene expression related to *Bcl2* gene for cell survival, *c-Myc* gene for cell proliferation, *MMPs* genes for migration/invasion, *FoxF1* gene for angiogenesis and *PROM1* for cancer stem cells.

When the HH signaling pathway is in its off state, PTCH destabilizes SMO and SMO activity is inhibited by the binding of PTCH (Figure 1a). GLI proteins bind to SUFU repressors and are then processed by proteasome. GLI could be completely degraded or generated as a N-terminal truncated GLI repressor (GLI^R^). GLI^R^ binds to HH target gene promoters and turns off their expression. In the on state, HH-producing cells release ligands in a spatially restricted manner, forming an HH protein gradient (Figure 1b) [14]. This process starts after an HH ligand binds to PTCH-1, and both of them are degraded in lysosomes. It could relieve the inhibition of SMO from PTCH, and then leads to the subsequent stimulation of G-protein-coupled receptor (GPCR)/SMO activity. GLI proteins are released from the inhibition of SUFU and are subsequently activated (GLI activated form, GLI^A^), which triggers the expression of downstream target genes and activates their cellular functions [7]. Transcriptional targets of the HH signaling pathway include genes in different categories: (1) cell survival and cell proliferation (*Bcl2*, *c-Myc*, *CDK* and *cyclin B1*); (2) epithelial–mesenchymal transition (EMT), invasion, migration and metastases (*Matrix metalloproteinases* (*MMPs*) and *SNAIL*); (3) angiogenesis (*Forkhead box F1* (*FoxF1*) and *morphogenic protein 4* (*BMP4*)); and (4) chemotherapy resistance and cancer stem cell formation (*PROM1* and CD133) [3].

## 3. Noncanonical Hh Signaling Pathway

**Figure 2:**
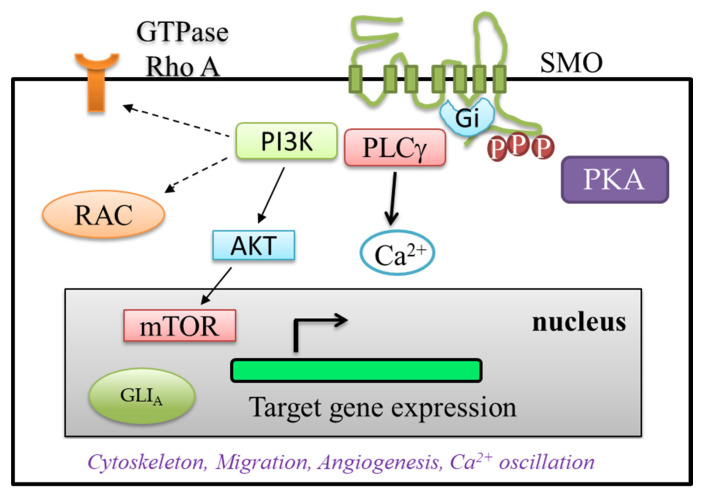
Noncanonical HH signaling pathway. SMO and GLI are activated through other signaling pathways such as PKA, GTPase, PI3K/mTOR or Rho to enable target gene expression. PKA phosphorylates the C-terminus of SMO at three sites. PI3K could activate the signaling through AKT, mTOR and turn on gene expression. PI3K could interact with RhoA and Rac, which could have effect on the cytoskeleton. PLCγ could act on Ca^2+^ flux. Therefore, noncanonical HH signaling pathway can regulate cytoskeleton, cell migration, angiogenesis and Ca^2+^ oscillation.

Broadly speaking, noncanonical HH signaling describes any pathway that involves HH elements but differs from the usual signaling pattern [2]. Noncanonical HH signaling involves SMO or GLI activation via other pathways (GTPase, PKA, Rho or phosphoinositide 3-kinase (P13K)/mTOR) (Figure 2). The noncanonical pathway acts as an alternative route when the canonical HH pathway fails to be activated. Therefore, noncanonical HH signal transduction could serve as an escape from the canonical HH signaling affected by cytotoxic or inflammatory stress [15].

SMO-dependent noncanonical pathway could control the metabolism [16]. SMO is a functional of G-protein-coupled receptors (GPCRs), including N-terminal cysteine rich domain (CRD), extracellular loops, seven transmembrane domain and an intracellular C-terminal domain [17]. SMOSmo- heterotrimeric G-inhibitory (Gi) family proteins coupling could regulate calcium (Ca^2+^) flux, RhoA and Rac activation (Figure 2) [18,19]. SMO activates these molecular switches and targets specific molecules that modulate noncanonical HH signaling responses [4]. Active Gαi can negatively regulate adenylyl cyclase, thereby inhibiting intracellular cAMP and PKA activity [5]. SMO-dependent, noncanonical HH signaling also elicits specific cellular responses via the activation of small G-proteins (GTPases). Small GTPases are monomeric G proteins that, acting as molecular switches, can regulate cellular function. Moreover, guanine-exchange factors (GEFs) activate small GTPase-bound guanosine-5′-triphosphate (GTP) when in the on state. GTPases are then inactivated by the hydrolysis of the bound GTP to guanosine diphosphate (GDP), an intrinsically slow process facilitated by GTPase-activating proteins (GAPs). 

SMO can be activated by some protein kinases in non-canonical pathways such as Rho, Rac, Src and PI3K/phospholipase C gamma (PLCγ), as well as secondary messengers such as calcium (Figure 2). It can affect cytoskeletal arrangement and cellular migration [4]. Small GTPases may be categorized into four families: Ras, Rho, Arf and Rab. The Rho family, which mediates cytoskeletal reorganization, can be further divided into three subfamilies: Rho, Rac and Cdc42. These subfamilies regulate cytoskeletal rearrangements through the polymerization of actin filaments, and different rearrangements allow the coordination essential to cell motility [4]. Therefore, SMO-dependent noncanonical HH signaling could affect cellular migration and contribute to the cancer progression [2].

## 4. SMO and Breast Carcinoma

Breast carcinoma is the most common cancer among women worldwide [20]. Classified based on the expression of hormone (estrogen or progesterone) receptors (HR) and human epidermal growth factor receptor 2 (HER2), breast cancer includes four subtypes: (1) HR+/Her2− (luminal A); (2) HR+/Her2+; (3) HR−/Her2+; and (4) triple-negative breast cancer (TNBC), which is negative for estrogen receptor (ER), progesterone receptor (PR) and Her2 [21]. TNBC is more aggressive than the other types and is associated with poor prognoses because it usually fails to respond to standard adjuvant therapy and exhibits cancer-stem-cell-like characteristics [22,23].

The HH/SMO signaling pathway plays an important role in breast cancer development, progression, invasion and metastasis [24,25,26]. The pathway regulates breast tumorigenesis affecting cell proliferation, self-renewal, maintenance of cancer stem cells and epithelial–mesenchymal transition (EMT) [27,28,29]. The activation of SMO plays an essential role in the development of dysplasia of the mammary ducts [29,30,31]. SMO exists in both ductal carcinoma in situ (DCIS) and invasive breast cancer (IBC) but is not present in normal tissues [30]. Overexpression of SMO is associated with tumor size, lymph node metastasis and postoperative recurrence [32]. Therefore, HH signaling pathway molecules could be indicators for recurrent breast carcinoma.

In ER+ subtype breast cancer, estrogen triggers the overexpression of SHH and GLI1. It activates SHH signaling and enhances cancer cell invasiveness of the ER-positive T47D (HER2−) and BT-474 (HER2+) cells [33]. There may be cross-talk between ER- and SHH-signaling pathways facilitating the invasiveness of ER-positive cancer cells [29]. Triple-negative breast cancer (TNBC) presents a moderate amount of basal-like progenitors that retain the primary cilia characteristics [34,35]. The SHH signaling pathway orchestrates the angiogenesis in TNBC [34]. Overexpression of Hedgehog molecules SMO and GLI1 exists in breast cancer and mammary hyperplasia, which can affect histological grade or tumor stage in TNBC [36]. In addition, the upregulation of HH pathway molecules were found in positive lymph nodes-positive breast cancer cases. The HH signaling pathway probably affects the activation of cancer stem cells and the progression, invasion and metastasis of TNBC. In in vivo studies, SHH overexpression facilitated the growth of orthotopic xenograft and the lung metastasis [37]. Canonical SHH signaling triggers angiogenesis of TNBC via metalloproteases (MMPs), cysteine-rich angiogenic inducer 61 (Cyr61, CCN1) and vascular endothelial growth receptor 2 (VEGFR2), enhancing growth and metastasis [29,34,37,38]. SHH pathway affects bone metastasis, with osteolysis in TNBC [29]. In TNBC cell line MDAMB231, the HH signaling pathway promotes the migration and invasion of breast cancer cells via carbonic anhydrase (CA) XII [39]. In in vitro studies, overexpression of SHH enhanced cell proliferation, colony formation, migration, and invasion of TNBC [37,40]. However, another study demonstrated that SMO expression did not correlate with patient age or metastasis, but correlated with earlier onset of TNBC [26]. 

The cancer microenvironment/stroma consists of endothelial cells, immune cells, adipocytes and cancer-associated fibroblasts (CAFs) [41]. CAFs fuel cancer cells via the secretion of soluble factors that trigger metastasis and chemoresistance [42,43,44,45]. The microenvironment of breast cancer is affected by the type II noncanonical SHH signaling pathway, which can enhance cancer development and metastasis [5,29,46]. This process includes extracellular acidification, inflammation and activation of matrix metalloproteases [42,47,48]. In such a microenvironment, the tumor-associated macrophages with aberrant genetic and epigenetic changes trigger overexpression of signaling molecules that prolong the tumor cells’ survival [49]. 

Inhibitors targeting the signaling pathway of SHH, Notch, cyclin-dependent kinases (CDKs), mTOR and WNT have become promising treatment strategies [49]. HH inhibitors may emerge as valuable therapeutic option in the future [34]. Ruiz-Borrego et al. used a combination of sonidegib (LDE225) (a small molecular, oral inhibitor of the SMO/SHH pathway) and docetaxel (a drug for metastatic breast cancer) to treat advanced TNBC in a phase Ib clinical trial study [50]. The results show one patient with a complete response and two patients with long-lasting stabilizations out of ten patients. According to Benvenuto’s study, a SMO inhibitor (GDC-0449) and GLI inhibitor (GANT-61) targeting the SHH/GLI pathway suppressed cell growth both in vitro and in vivo [51]. Therefore, downstream SMO targeting seems to be superior to upstream SMO targeting in interrupting the HH signaling in breast cancer [51]. Cyclopamine could directly bind to SMO and regulates the expression of Hedgehog molecules SHH, PTCH1, GLI1 and GLI2. It could decrease growth of human breast cancer cells [52]. Breast cancer cells-condition medium with cyclopamine could interfere osteoclast activity [53]. SMO inhibitor cyclopamine decreased SMO, GLI and CD44 expression and reduced cell proliferation of breast cancer stem cells for chemoresistance [27,54,55,56]. Cyclopamine significantly reduced the invasiveness and estrogenic potency in breast cancer [57]. Therefore, targeting SMO could be an effective way to treat breast cancer.

## 5. SMO and Liver Cancer

Hepatocellular carcinoma remains one of the leading causes of cancer-related death in Asian countries [58]. HCC is the most common primary liver cancer, comprising 80% of cases [59]. The causes of liver fibrosis to HCC can include the responses to viral hepatitis, alcohol, steatosis, autoimmune diseases, etc. [60,61,62,63]. These factors can induce a harmful inflammatory reaction and repeated chronic liver injury, eventually resulting in hepatocarcinogenesis [60,64]. Surgical resection and liver transplantation (LT) remain the mainstay treatment for HCC [65,66]. However, HCC has a 50–75% five-year recurrence rate after the surgery [67]. 

The Hedgehog signaling pathway is highly activated in HCC patients [68,69]. It plays a role in hepatocarcinogenesis, invasiveness, recurrence and HCC cancer stem cells [70,71]. Transformation of HBx (HBV gene product HBx protein) can activate the HH signaling pathway. SMO is an important regulator in the repair of adult liver tissue and plays a key role in the promotion of epithelial–mesenchymal transition (EMT) during early hepatocarcinogenesis [72]. SMO expression in primary hepatocytes may be upregulated after Fas-induced liver injury and holds potential value as a prognostic factor in HCC patients [73]. Overexpression of SMO induces the expression of c-Myc, which plays a significant role in hepatocarcinogenesis and SMO overexpression is correlated with tumor sizes [74]. Overexpression of HH signaling molecules predicts a higher risk of postoperative HCC recurrence [68]. The activation of HH signaling enhances the G2–M transition following overexpression of cyclin B1 and cyclin-dependent kinase 1 (CDK1), facilitating cell proliferation [75]. Moreover, the overexpression of SMO mRNA is present in cancer stem cell CD133+ mouse liver cell line Hepa1-6 [76]. In addition, SMO polymorphisms in transplant recipients may increase the risk of HCC recurrence following liver transplantation [77]. This evidence could be clinically valuable when determining the prognoses of HCC cases. The C-terminal lysine mutation (K575M) in SMO can affect the binding between SMO and PTCH, and is able to release SMO from PTCH inhibition [70].

The SMO inhibitor cyclopamine has been shown to reduce DNA synthesis, resulting in inhibition of the cell growth, invasiveness, and motility of HCC cells [78]. In addition, cyclopamine suppresses cell viability and increases apoptosis after downregulating Bcl-2 in HCC cells [73]. Sicklick et al. also found that 3-keto-N-aminoethylcaproyldihydrocinnamoyl cyclopamine (KAAD-cyclopamine) can inhibit HH signaling activity and expression of Myc, as well as reducing the growth rate of Hep3B cells [74]. Kim et al. reported that HCC cells harboring SMO mutations are otherwise unresponsive to KAAD-cyclopamine [79]. The administration of SMO antagonist GDC-0499 resulted in the inhibition of hepatocarcinogenesis in HBx transgenic mice [80]. Jeng et al. reported that cyclopamine or GDC-0499 decreased expression of HH genes and reduced HCC cell growth in a mouse model [81,82]. Moreover, GDC-0449 reduced the cell migration, invasion and metastasis to lung of chondroitin sulfate synthase 1 (CHSY1)-induced HCC cells [83]. In a Phase I study, the pharmacokinetics and safety of GDC-0449 was evaluated in patients with HCC or hepatic impairment. However, the results are difficult to tell the adverse events from advanced HCC or GDC-0449 exposure [84]. Further study is required to verify clearly how to regulate HH signaling mitigate HCC progression with minor adverse events [85]. Overall, there is a consensus that SMO inhibitors may represent a potentially beneficial strategy against hepatocarcinogenesis [86]. 

## 6. SMO and Pancreatic Cancer

Pancreatic cancer is one of the most highly invasive of the solid cancers and actively communicates with the desmoplastic stroma [87]. The aberrant expression of SHH is correlated with oncogenic Kras, which is highly mutated in pancreatic ductal adenocarcinoma (PDAC) [88]. It has been shown that Shh is a target gene of NF-κB, which is constitutively active in pancreatic cancer [89]. Accordingly, both canonical and noncanonical HH signaling are present in the tumor cells, but ligand-dependent HH signaling mainly exists only in stromal cells [90]. SMO plays an important role in the development of pancreatic cancer cell metastasis [91]. It has also been shown that SMO is upregulated in cancer-associated fibroblasts (CAF), the predominant stromal cell type, comparing with normal pancreatic fibroblasts [92]. Hypoxia found in pancreatic ductal adenocarcinoma increased the transcription of SHH, SMO and GLI-1 and activated the SHH pathway to promote invasiveness [93]. Meanwhile, tumor necrosis factor-alpha and interleukin-1 beta in the hyperplasia stroma enhanced the carcinogenesis of pancreatic ductal adenocarcinoma via activation of the HH pathway [94]. The knockdown of SMO could inhibit pancreas cancer cells in terms of self-renewal, epithelial–mesenchymal transition (EMT), invasion, migration, lung metastasis, chemoresistance to gemcitabine and development of pancreatic cancer stem cells [95]. SMO regulated EMT, invasion and migration of pancreatic cancer stem cells [95]. Thus, the dysregulated SMO in pancreatic cancers could be a therapeutic target [96].

A novel GDC-0449 analog was used to decrease side effects in pancreatic cancer treatment [91]. AZD8542, a novel HH antagonist, inhibited the progression of pancreatic cancer with an emphasis on the role of the stroma compartment [97]. The ablation of the SMO gene in stromal fibroblasts caused increased proliferation of pancreatic tumor cells and the activation of oncogenic protein kinase B (AK1) in fibroblasts [98]. A SMO inhibitor increased the intratumoral vasculature [99]. In a mouse model, this inhibition facilitated the delivery of chemotherapy drugs in treating pancreatic cancer [99]. GDC-0449 has been shown to downregulate HH signaling and to decrease fibroblast-induced doxorubicin resistance [100]. Moreover, the genetic ablation of SMO in stromal fibroblasts in a Kras G12D mouse model disrupted paracrine HH signaling and increase acinar-ductal metaplasia [101]. Fibroblasts with SMO deletion exhibited overexpression of transforming growth factor-alpha (TGF-α), leading to the activation of epidermal growth factor receptor signaling in acinar cells [101]. 

## 7. SMO and Colon Cancer 

Colon cancer is one of the most common gastrointestinal cancers worldwide [102]. Colorectal cancer is the second most common cause of cancer death in the United States. SMO affects colon cancer progression and can act as a biomarker for liver metastasis [103]. Increased SMO expression was found in colon cancer tissues compared to normal tissues via immunohistochemistry staining. The level of SMO expression is correlated with metastasis and T stage. In addition, SMO expression in colorectal cancer correlates with patients’ outcome [104]. Colon cancer presents a heterogeneous tumor type with a subpopulation of cancer stem cells. WNT and HH signaling components are increased in cancer stem cells according to whole-transcriptome analysis [105]. In colon tissue, the ratio of SMO and GLI protein expression is increased significantly in cancer and adenoma tissue compared with normal colon tissue [106]. However, some studies still question the exact role of the HH signaling in the carcinogenesis and progression of colon cancer [107,108,109]. The mutations of the SMO protein (A324T, V404M and T640A) in colon cancer produced no aberrant HH signaling activity [107]. Chatel et al. showed that the expression of the HH pathway members was impaired in colon cancer cell lines [108]. Although SHH was upregulated, Gerling et al. demonstrated that the downstream activity of HH signaling decreased in colon cancer [109]. Activation of stromal HH was able to suppress a colonic tumor via modulating BMP signaling and restricting colonic stem cells [109].

Despite these controversies, most investigators agree that SMO is a potential target for colon cancer treatment [39]. SMO inhibitor GDC-0449 suppresses colon cancer cells proliferation and triggers apoptosis via the downregulation of Bcl-2 [110]. GDC-0499 is used to inhibit and modulate cellular plasticity and invasiveness in colorectal cancer [111]. Therefore, SMO could be a potential treatment target for colon cancer [96]. 

## 8. Pharmacological Studies of SMO Inhibitors in Other Cancers

Based on the results described above, SMO-related inhibitors have shown anti-cancer ability in vitro and in vivo, even in clinical trials (Table 1). Many SMO inhibitors could bind to the seven-transmembrane of SMO and were under investigation for clinical application [112]. Vismodegib (GDC-0499, ERIVEDGE^TM^), erismodegib (LDE-225, sonidegib) and glasdegib have been approved by the Food and Drug Administration (FDA) for treatment of basal cell carcinoma. Vismodegib has been used as a monotherapy or in combination with some chemotherapeutics in the clinical trials for the treatment of medulloblastoma, meningioma, glioblastoma, small-cell cancer, metastatic prostate cancer, metastatic pancreatic cancer, etc. [113]. However, vismodegib in combination with gemcitabine was not superior to gemcitabine alone in clinical trials with metastatic pancreatic adenocarcinoma patients [114]. Another clinical trial suggested that a benefit of vismodegib in combination with either Folinic acid, Fluorouracil, Oxaliplatin (FOLFOX) or Folinic acid, 5-FU, IRInotecan (FOLFIRI) was not found in colorectal cancer [115].

Erismodegib (LDE-225, sonidegib), another SMO antagonist, influences cancer stem cell activity and decreases the invasiveness of glioblastoma, renal-cell cancer and prostate cancer [121,122,123]. Saridegib (IPI-926), a modified form of cyclopamine, increased the delivery of gemcitabine to pancreatic ductal cancer in a mouse model [99]. It can potentially inhibit lung tumor and cholangiosarcoma xenografts [124,125]. CUR6414 directly binds to SMO to treat basal cell carcinoma [105], while BMS-833923 directly binds to SMO, reducing the growth of medulloblastoma, pancreatic cancer and cholangiocarcinoma in xenograft mice [116,126]. PF-5274857, a selectively potent SMO antagonist, can penetrate the blood–brain barrier to treat brain tumors or metastasis [127]. TAK-441, an oral SMO inhibitor, suppressed medulloblastoma and pancreatic cancers in mice, as well as mitigated the progression of prostate cancer in mouse xenograft models [119,128]. 

For acute myelogenous leukemia (AMC), the combined use of a SMO inhibitor LDE225 (sonidegib) or PF-04449913 (glasdegib) with the conventional drugs were demonstrated [117]. Two possible mechanisms have been proposed: direct affecting the intracellular pathway and indirect overcoming the drug resistance. Such combined therapy paves an innovative strategy for treatment of AML [111]. In a Phase I study of PF-04449913, 100 mg was a safe dose in Japanese patients with advanced hematologic malignancies [120]. SMO-related inhibitors could have anti-cancer ability in vitro or in vivo, even in clinical trials (Table 1). Three drugs, GDC-0449, LDE225 and PF-04449913 (Glasdegib), are FDA-approval drugs for basal cell carcinoma. 

However, the resistance to SMO inhibitors remains a challenge. The resistance could be from SMO mutation, SUFU deletion, GLI-2 amplification or other mechanisms [129]. Mutations of D473G or W533L of the SMO receptor could lead to the resistance to vismodegib by reducing the binding affinity [130]. Many smo-related drugs (GDC-0449, erismodegib, saridegib, ZSP1-1602, NVP-LEQ-506, glasdegib and taladegib) are still under trials, and the studies of possible side effects are ongoing (Table 2). GDC-0449 has had several Phase 1, 2 and 4 clinical trials with different indications, such as solid tumors, pancreatic cancer, medulloblastoma and metastatic BCC. The next generation of novel SMO inhibitors must overcome the obstacle/resistance of SMO mutations [113,131].

## 9. Using SMO Antagonists to Inhibit Cancer Stem Cells

Cancer stem cells (CSC) are a subpopulation of cancer cells that retain the characteristics of self-renewal and self-sustenance [131]. They are usually involved in development, progression, recurrence and metastasis of tumors. They also contribute to drug resistance in chemotherapy [131,132]. SHH/SMO/GLI affects EMT to induce the polarized epithelial cells transformation with active motility. Such cells trigger the invasiveness and metastasis of cancer [105,132]. 

SHH/SMO signaling pathway activates in cancer stem cells (CD133+) of the mouse hepatoma cell line Hepa1-6 [86]. CD133+ HCC cells with upregulated SMO mRNA have significantly higher colony proliferation and clonogenicity than CD133- HCC cells [76]. BMS-833923, a SMO inhibitor, significantly inhibits osteoblast differentiation of human mesenchymal stem cells (hMSCs) causing in a decrease of alkaline phosphate activity and a decrease of osteoblast-related gene expression and in vitro mineralization [133]. CD44 with overexpression of HH/SMO pathway genes and some self-renewal marker proteins (SOX2, OCT4 and NANOG) in several gastric cancer cell lines were found [134]. SMO shRNA or inhibitors can significantly suppress the spheroid formation and tumor growth of gastric cancer cell lines. Furthermore, HH/SMO inhibition could be helpful to reverse the chemoresistance of CD44+ spheroid gastric cancer cells to 5-fluorouracil and cisplatin [134].

There are some proposals of mechanisms of cancer stem cell formation. Genetic mutations induced by endogenous or exogenous stimuli transform adult stem cells into cancer stem cells [135,136,137,138]. The main signaling pathways involved include Hedgehog, Wnt, Notch, BMP, Bmi, PI3K/Akt, etc. [139]. Many lines of evidence support the idea that SHH signaling is important in maintaining cancer stem cell in various cancers [12,140,141,142]. Neoplasms with activated SHH signaling pathway in cancer stem cells consist of glioblastoma, chronic myeloid leukemia, multiple myeloma, hepatocellular carcinoma and cancers of the colon, breast and pancreas [7]. 

Drug resistance develops following SHH/SMO/GLI signaling, upregulating drug-transport-pump expression in cancer stem cells [131]. SHH/SMO inhibitors have been shown to inhibit the CSCs of some cancers, including pancreatic cancer (ALDH+ cells), colon cancer (CD133+ cells), breast cancer (CD44 +CD24− cells) and gastric cancer (CD44+ cells) [143,144,145,146]. Combining SHH/SMO/GLI inhibitors and chemotherapy, radiation therapy, or immunotherapy to target CSCs has become a promising treatment [132]. A SMO inhibitor, 2-chloro-N1-[4-chloro-3-(2-pyridinyl)phenyl]-N4,N4-bis(2-pyridinylmethyl)-1,4- benzenedicarboxamide (MDB5) (an analog of GDC-0449), seemed to be more effective than GDC-0449 in treatment of pancreatic CSC M1A PaCa-2 cells [91]. MDB5 downregulated ALDH1, CD44, Oct-3/4 (key tumor markers of pancreatic CSC), Bcl-2, GLI-1 and SHH and upregulated Bax. In ER-positive breast cancer cells remodeling of the cancer microenvironment could facilitate an antioxidant response to SHH signaling to enhance the CSC activity [147].

SMO inhibitor vismodegib (GDC-0449) significantly suppressed cell proliferation, cell invasion and mammosphere formation of a TNBC stem-cell line [135]. It also inhibited the protein expression and phosphorylation of downstream signaling molecules to induce cell apoptosis. In a xenograft mouse model, pretreatment of HCC1806 cells (a TNBC stem cell line), with vismodegib significantly inhibited tumor growth [135]. This evidence shows that SMO antagonists can target breast CSCs. This has a potential as a promising strategy in clinical applications for TNBC [148]. 

## 10. Conclusions

**Figure 3:**
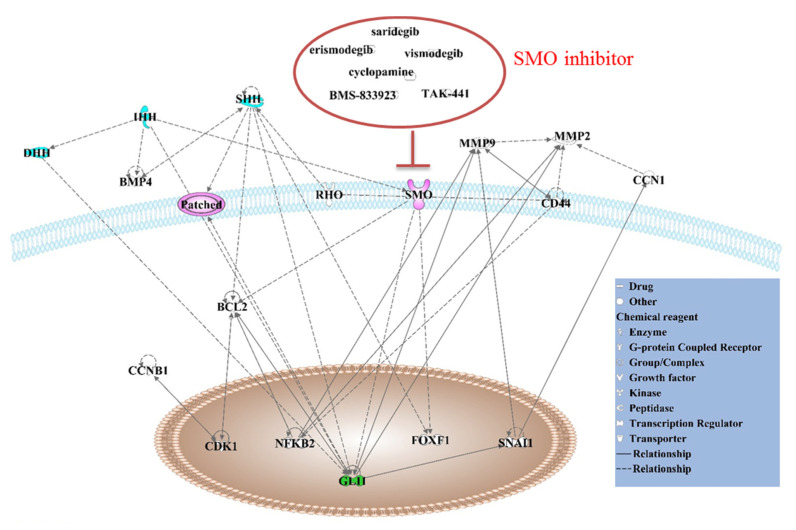
SMO in cancer. Major Hedgehog signaling pathway molecules, HH ligands, Patched, SMO and GLI, are labeled in color. SMO inhibitors include vismodegib (GDC-0449), cyclopamine, TAK-441, etc. Molecules (MMP2, Rho, FoxF1, Bcl2, NFKB, etc.) interact with Hedgehog molecules; the direct interactions between molecules are shown with solid lines and the indirect relationships between molecules are shown with dotted lines. The figure was plotted using Ingenuity Pathway Analysis software.

In cancer, the Hedgehog molecule SMO interacts directly or indirectly with several molecules, including MMPs, BMP4, Rho, CCN1, etc. (Figure 3). SMO antagonists such as vismodegib, cyclopamine, erismodegib, saridegib, BMS-833923 and TAK-441 have been identified (Figure 3). SMO antagonists have been approved for clinical use or clinical trials in treating a variety of cancers (Table 2) [149]. SMO seems to be an important drug target, with a deep, pocket-like structure that allows efficient and selective drug binding. In addition, SMO inhibitors serve as another strategy against cancer stem cells [150]. Thus, SMO represents a promising therapeutic target for the inhibition of HH signaling in the treatment of a spectrum of malignancies [151]. The current clinical impact of SMO antagonists has been emphasized recently in cancer therapy, especially for a variety of solid tumors [7]. Such treatments could be beneficial to patients, either with a single use or as an adjuvant or adjunct to conventional chemotherapy [132].

The intra-tumor heterogeneity needs to be taken into consideration when considering cancer therapies, because this heterogeneity can contribute to tumor progression [152]. Heterogeneity also increases the difficulty of cancer treatment. More importantly, some mutations can lead to the resistance to SMO antagonists. SMO mutations that impair drug binding to SMO can occur at multiple levels [153]. It is necessary to discover new SMO antagonists [154]. Combined use of drugs to target different components at different levels of the HH pathway may be able to improve the issue of drug resistance [154]. Further study of the structural analogs and detailed mechanisms of hedgehog inhibitors, including the noncanonical pathway, is needed. The strategy for Hedgehog inhibitor alone or combination with other anticancer drug needs to overcome known drug resistance and adverse events [155]. A better understanding of the HH/SMO pathway could be useful for developing a new class of clinically efficient drugs.

## Figures and Tables

**Table 1:** Hedgehog/Smo drugs in different cancer types

Cancer Type	Treatment	Level of Evidence	References
Breast cancer	LDE-225 combined with docetaxel	Phase 1b	[50]
	GDC-0449	in vitro and in vivo	[51]
	Cyclopamine	In vitro	[27,54,55,56,57]
Liver cancer	Cyclopamine	in vitro and in vivo	[73,79,83]
	KAAD-cyclopamine	in vitro	[74]
	GDC-0499	in vitro and in vivo	[80,81,83]
Pancreatic cancer	MDB5	in vitro and in vivo	[91]
	AZD8542	in vivo	[97]
	IPI-926 (saridegib)	in vivo	[100]
	GDC-0449	in vivo	[99]
	BMS-833923	in vivo	[116]
	TAK-441	in vivo	[117]
Colon cancer	GDC-0449	in vitro	[110,111]
Basal cell carcinoma	GDC-0499	FDA approved	
	LDE-225	FDA approved	
	PF-04449913 (glasdegib)	FDA approved	
	CUR6414	in vivo	[118]
Medulloblastoma	BMS-833923	in vivo	[116]
	TAK-441	in vivo	[119]
Acute myelogenous leukemia	PF-04449913 (glasdegib)	Phase 1	[120]

Mechanism: SMO inhibitors LDE225, GDC-0449, cyclopamine, KAAD-cyclopamine, IPI-926, BMS-833923 and PF-04449913 (glasdegib) could bind to 7TM domain of SMO.

**Table 2:** SMO drug in clinical trials (last updated in 2020, Jan-July)

Drug	Indication	FDA Approval Status	Trial Status	NCT#
GDC-0449	plasma-cell myeloma, metastatic solid tumor, B-cell non-Hodgkin lymphoma	Phase 2	Recruiting	NCT03297606
	tumor, neoplasoa, cancer	Phase 2	Not yet recruiting	NCT04341181
	lymphoma, advanced solid tumor, advanced multiple myeloma	Phase 2	Recruiting	NCT02465060
	cancer	Phase 2	Recruiting	NCT03498521
	advanced chondrosarcoma	Phase 2	Active, not recruiting	NCT01267955
	metastatic pancreatic cancer or solid tumors	Phase 1	Active, not recruiting	NCT00878163
	grade 4 astrocytoma	Phase 1/Phase 2	Recruiting	NCT03158389
	primitive neuroectodermal tumor, medulloblastoma	Phase 2	Recruiting	NCT01878617
	solid tumor, glioblastoma, plasma cell myeloma, ovarian cancer, metastatic solid tumor, B-cell non-Hodgkin lymphoma	Phase 2	Recruiting	NCT02925234
	metastatic basal-cell carcinoma	Phase 4	Recruiting	NCT03610022
Glasdegib	acute myeloid leukemia	Phase 3	Recruiting	NCT03416179
	chronic/acute myelomonocytic leukemia	Phase 2	Active, not recruiting	NCT02367456
	chronic myelomonocytic leukemia, myelodysplastic syndrome	Phase 2	Active, not recruiting	NCT01842646
	myelodysplastic syndrome	Phase 2	Active, not recruiting	NCT02367456
	glioblastoma	Phase 1/2	Recruiting	NCT03466450
	relapsed acute myeloid leukemia	Phase 1/2	Recruiting	NCT03390296
	acute myeloid leukemia	Phase 2	Completed	NCT01546038
	acute myeloid leukemia	Phase 2	Recruiting	NCT04051996
	acute myeloid leukemia with myelodysplasia-related changes	Phase 2	Recruiting	NCT04231851
LDE225/erismodegib	solid tumor, pancreatic adenocarcinoma, non-small cell lung cancer, colorectal cancer, metastatic urothelial carcinoma, metastatic solid tumor, metastatic pancreatic adenocarcinoma, metastatic melanoma, metastatic gastric adenocarcinoma, metastatic colorectal cancer, malignant urothelial neoplasm, head and neck squamous cell carcinoma	Phase 1	Recruiting	NCT04007744
	medulloblastoma	Phase 1	Recruiting	NCT03434262
	basal cell carcinoma	Phase 1	Completed	NCT00880308
Saridegib	basal-cell nevus syndrome	Phase 3	Not yet recruiting	NCT04308395
	basal-cell carcinoma	Phase 2	Recruiting	NCT04155190
Taladegib	gastroesophageal junction adenocarcinoma	Phase 1/Phase 2	Active, not recruiting	NCT02530437
	malignant solid tumor, metastatic lymphoma, advanced colon cancer, advanced breast cancer, cholangiocarcinoma, metastatic refractory colon cancer, metastatic soft tissue sarcoma	Phase 1	Completed	NCT02784795
NVP-LEQ-506	advanced solid tumor	Phase 1	Completed	NCT01106508
ZSP-1602	advanced solid tumor, glioblastoma, basal-cell carcinoma, neuroendocrine tumor, gastroesophageal junction adenocarcinoma, medulloblastoma, small-cell lung cancer	Phase 1	Recruiting	NCT03734913

## Abbreviations

SMO	Smoothened
HH	Hedgehog
mTOR-Akt	Mammalian target of rapamycin-protein kinase B
PKA	Protein kinase A
SUFU	Suppressor on fused homolog
GPCR	G-protein-coupled receptor
EMT	Epithelial–mesenchymal transition
MMPs	Matrix metalloproteinases
FoxF1	Forkhead box F1
BMP4	Morphogenic protein 4
PI3K	Phosphoinositide 3-kinase
CRD	Cysteine rich domain
Gi	G inhibitory
GEFs	Guanine exchange factors
GTP	Guanosine-5′-triphosphate
GDP	Guanosine diphosphate
GAPs	GTPase-activating proteins
PLCγ	Phospholipase C gamma
HR	Hormone receptor
HER2	Human epidermal growth factor receptor 2
TNBC	Triple negative breast cancer
ER	Estrogen receptor
PR	Progesterone receptor
DCIS	Ductal carcinoma in situ
IBC	Invasive breast cancer
Cyr61	Cysteine-rich angiogenic inducer 61
VEGFR2	Vascular endothelial growth receptor 2
CA	Carbonic anhydrase
CAFs	Cancer-associated fibroblasts
CDKs	Cyclin-dependent kinases
LT	Liver transplantation
HBx	HBV gene product HBx protein
CDK1	Cyclin-dependent kinase 1
CHSY1	Chondroitin sulfate synthase 1
PDAC	Pancreatic ductal adenocarcinoma
TGF-α	Transforming growth factor-alpha
FDA	Food and Drug Administration
FOLFOX	Folinic acid, Fluorouracil, Oxaliplatin
FOLFIRI	Folinic acid, 5-FU, IRInotecan
AMC	Acute myelogenous leukemia
CSC	Cancer stem cell
hMSCs	Human mesenchymal stem cells
